# *In vitro* Assessment of the Probiotic Properties and Bacteriocinogenic Potential of *Pediococcus pentosaceus* MZF16 Isolated From Artisanal Tunisian Meat “Dried Ossban”

**DOI:** 10.3389/fmicb.2018.02607

**Published:** 2018-11-09

**Authors:** Mohamed Zommiti, Emeline Bouffartigues, Olivier Maillot, Magalie Barreau, Sabine Szunerits, Khaled Sebei, Marc Feuilloley, Nathalie Connil, Mounir Ferchichi

**Affiliations:** ^1^Unité de Protéomique Fonctionnelle et Potentiel Nutraceutique de la Biodiversité de Tunisie, Institut Supérieur des Sciences Biologiques Appliquées de Tunis, Université de Tunis El-Manar, Tunis, Tunisia; ^2^Laboratoire de Microbiologie Signaux et Microenvironnement EA 4312, Université de Rouen, Normandie Université, Évreux, France; ^3^Univ. Lille, CNRS, Centrale Lille, ISEN, Univ. Valenciennes, UMR 8520, IEMN, Lille, France; ^4^College of Applied Medical Sciences, Clinical Laboratory Department, King Faisal University, Al-Ahsa, Saudi Arabia

**Keywords:** Tunisian fermented meat, Dried Ossban, *Pediococcus pentosaceus*, probiotic, pediocin MZF16

## Abstract

*Pediococcus pentosaceus* MZF16 has been isolated from artisanal Tunisian meat so called “Dried Ossban,” an original ecological niche, and identified by MALDI-TOF mass spectrometry and 16S rDNA sequencing. This bacterium showed a high tolerance to gastric stress conditions, and toward bile salts. *P. pentosaceus* MZF16 also demonstrated a hydrophobic surface profile (high adhesion to xylene), autoaggregation, and adhesive abilities to the human intestinal Caco-2/TC7 cell line. These properties may help the bacterium colonizing the gut. Furthermore, MZF16 was found to be resistant to gentamycin and chloramphenicol but did not harbor any transferable resistance determinants and/or virulence genes. The data also demonstrated absence of cytotoxicity of this strain. Conversely, *P. pentosaceus* MZF16 can slightly stimulate the immune system and enhance the intestinal epithelial barrier function. Moreover, this bacterium has been shown to be highly active against *Listeria* spp. due to bacteriocin production. Characterization of the bacteriocin by PCR amplification, sequencing and bioinformatic analyses revealed that MZF16 produces a bacteriocin 100% identical to coagulin, a pediocin-like inhibitory substance produced by *Bacillus coagulans*. To our knowledge, this is the first report that highlights the production of a pediocin 100% identical to coagulin in a *Pediococcus* strain. As coagulin, pediocin MZF16 has the consensus sequence YYGNGVXCXXXXCXVXXXXA (X denotes any amino acid), which confirms its belonging to class IIa bacteriocins, and its suitability to preserve foods from *Listeria monocytogenes* development. According to these results, *P. pentosaceus* MZF16 can be proposed as a probiotic and bioprotective agent for fermented foods, including Tunisian dry meat and sausages. Further investigations will aim to study the behavior of this strain in meat products as a component of functional food.

## Introduction

Lactic Acid Bacteria (LAB) are widely present in foods and used as biopreservatives, in addition to scent, texture, and flavor enhancers. Various investigations have demonstrated the tight connection between these properties and the ability of the LAB to produce, via fermentation, a wide range of sugars and metabolites such as acids (lactic and acetic acid), acetone, ethanol, exopolysaccharides, diacetyl, some specific proteases, and proteinaceous antimicrobials also known as bacteriocins (Papagianni, 2012; Cotter et al., 2013; Gaspar et al., 2013; Saad et al., 2013; Mazzoli et al., 2014; Gudiña et al., 2015; Barbosa et al., 2017). Besides, some LAB are also known as probiotics. According to Hill et al. (2014), probiotics are defined as live microorganisms that when consumed in adequate amounts exert beneficial effects in the host with a GRAS status (Generally Regarded As Safe). Probiotic strains mainly include members of the genera *Lactobacillus* and *Bifidobacterium*, but bacteria belonging to the genus *Pedioccocus* have also been tested and already used (Vidhyasagar and Jeevaratnam, 2012; Martino et al., 2013; Belhadj et al., 2014; Savedboworn et al., 2014; Dubey et al., 2015; Chen et al., 2017). Such investigations have led to an obvious support of the significance of *Pediococci* in the field of probiotics, indicating that new isolated strains from various food matrices and belonging to this genus may play a key role to be used in new generation of functional foods. The list of probiotics needs to be urgently expanded due to the non-stop demand for safe foods by the consumers, and since every single species of LAB has its unique criteria and properties leading to a potential use for different purposes. The biosafety use of *Pediococcus* spp. as a probiotic requires specific studies that demonstrate a safety evaluation of microorganisms throughout the food chain, search for virulence factors, absence of acquired antibiotic resistance and probiotic viability at the end of shelf life for every single strain that claims GRAS or QPS (Qualified presumption of safety) status.

According to Semjonovs and Zikmanis (2008), *Pediococci* correspond to a group of Gram positive, coccus shaped, non-motile, non-spore forming, homofermentative bacteria, which are involved in the manufacturing of fermented foods at industrial level. The genus *Pediococcus* spp. includes several species encompassing *Pediococcus inopinatus, P. dextrinicus, P. claussenii, P. damnosus, P. cellicola, P. ethanolidurans, P. parvulus, P. stilesii, P. acidilactici*, and *P. pentosaceus* (Dobson et al., 2002; Holzapfel et al., 2006; Todorov and Dicks, 2009; Holzapfel and Wood, 2014). The number of investigations involving the *Pediococcus* genus has been continuously increasing, particularly with *P. acidilactici* and *P. pentosaceus*, studying their genetic, molecular and physiological aspects. Both species are massively used in industry in purpose to ferment foods such as vegetables, sausages and meat derivatives due to the preservative properties of pediocin (Papagianni and Anastasiadou, 2009). Numerous studies have reported that strains of *P. pentosaceus* are frequently isolated from various food sources and biotopes encompassing plant materials, bacterial ripened cheese, beverages, pickles, wine, dairy, and meat products, with a potent role as starter cultures involving in the manufacturing of fermented foods (Halami et al., 2005; Midha et al., 2012; García-Ruiz et al., 2014; Lv et al., 2014; Carafa et al., 2015; Ilavenil et al., 2016). According to the bacteriocin classification proposed by Cotter et al. (2005), pediocins represent biomolecules that can be synthesized by several LAB and present a broad spectrum of antimicrobial activity against Gram-positive bacteria (Papagianni and Anastasiadou, 2009). It is noteworthy that pediocins may exert their antimicrobial potential even at nanomolar levels (Papagianni, 2003). Pediocins are highly active against pathogenic bacteria, particularly *Listeria monocytogenes*. The presence of this sturdy pathogen in dairy products, sausages and vegetables can be harmful to immune-compromised patients and pregnant women (Swaminathan and Gerner-Smidt, 2007) causing a serious disease called listeriosis leading to imminent mortality in vulnerable infected patients (Schuppler and Loessner, 2010). The most known pediocins are pediocin PA-1 from *P. acidilactici* PAC1.0 (Marugg et al., 1992) and pediocin AcH from *P. acidilactici* H (Motlagh et al., 1992) belonging, both, to class IIa bacteriocin. Another pediocin, pediocin PD-1, produced by *P. damnosus* (Bauer et al., 2005), is homologous to class I bacteriocins, which are lantibiotics such as actagardine and mersacidin. A class III heat labile bacteriocin of 80 kDa, the pediocin A has been characterized in *P. pentosaceus* FBB61 (Piva and Headon, 1994). *P. pentosaceus* and *P. acidilactici* correspond to the main species used in (i) pediocin production, (ii) fermentation processes as a starter culture for avoiding contamination, and (iii) probiotic supplements for humans and animal feeds.

In this context, the aim of the current study was to appraise the probiotic properties and bacteriocinogenic potential of *P. pentosaceus* MZF16, a new strain isolated from artisanal Tunisian meat called “Dried Ossban,” an original biotope recently cited for the first time in a research investigation led by Zommiti et al. (2018).

## Materials and methods

### Sampling collection and isolation of lactic acid bacteria (LAB)

About 40 samples of “Dried Ossban,” obtained from homemade production of fermented meat were sterilely collected from different governorates covering almost all the Tunisian territory, and analyzed in the laboratory (Zommiti et al., 2018). LAB strains were isolated from this original biotope so called “Dried Ossban,” a Tunisian traditional dry fermented meat typically prepared from sheep intestine and meat mixed with salt and spices and dried through exposure for several days to sun. Following sampling, 10 g was suspended in 90 mL of sterile peptone saline water, homogenized exhaustively. Appropriate decimal dilutions were plated onto de Man Rogosa Sharpe (MRS) agar and incubated at 37°C for 24–48 h. Colonies were randomly selected from MRS agar, examined for morphology (color, shape, elevation, density), Gram staining, and catalase production. Only Gram-positive and catalase-negative isolates including an isolate named MZF16 were retained and stored at −80°C in MRS broth containing 20% glycerol.

### Identification of *Pediococcus pentosaceus* MZF16 by matrix-assisted laser desorption ionization-time of flight mass spectrometry (MALDI-TOF MS) and 16S rDNA sequencing

The MZF16 strain isolated from “Dried Ossban” was identified by analysis of its total proteome, using an Autoflex III Matrix-Assisted Laser Desorption/ Ionization-Time-Of-Flight mass spectrometer (MALDI-TOF MS) (Bruker, Marcy-l'Etoile, France) coupled to the MALDI-Biotyper 3.1 system (Hillion et al., 2013; Zommiti et al., 2018). The result was expressed as a log (score) value, computed by comparison of the peak list of MZF16 strain with the reference main spectral pattern (MSP) in the database. A log (score) value ≥2.0 is the set threshold for a match at the species level (Sogawa et al., 2011).

Simultaneously to MALDI-TOF MS analysis, 16S rDNA sequencing was also used to identify the MZF16 strain. For this, total genomic DNA was extracted from overnight culture in MRS broth with GeneJET Genomic DNA Purification Kit (Thermo Scientific, France), and the 16S rRNA gene was amplified using the universal bacterial 16S rRNA gene primers 63F (5′-CAGGCCTAACACATGCAAGTC-3′) and 1492R (5′-GTTACCTTGTTACGACTT-3′) (Marchesi et al., 1998).

The PCR reaction was performed with the Thermo Scientific PCR Master Mix, in a total volume of 50 μL, using ~1 μM of forward and reverse primers and 10 pg−1 μg of DNA template, according to the manufacturer's instructions. The cycling parameters were an initial denaturing step at 95°C for 5 min, followed by 30 cycles of denaturing at 95°C for 1 min, annealing at 55°C for 1 min, and extension at 72°C for 1 min, then a final extension step at 72°C for 10 min. The amplicon obtained was sequenced by GENEWIZ (Takelay, United Kingdom) and BLAST-searched against the NCBI database to identify homologous sequences.

Following this identification step, the *P. pentosaceus* MZF16 strain was selected for further study and subjected to physiological tests for evaluating its probiotic attributes and bacteriocinogenic capacity.

### Growth in harsh conditions of salinity and temperatures

The ability of *P. pentosaceus* MZF16 to grow in harsh conditions has been carried out according the protocol of Abbasiliasi et al. (2012) with slight modifications. The growth potential of this bacterial strain in high salinity levels has been studied by the application of an ascendant gradient of NaCl concentration. The MZF16 strain was inoculated (1% v/v) into MRS broth with various concentrations of NaCl [w/v] (0.5, 2, 4, 6.5, and 9.5%) and bromocresol purple indicator, then incubated at 37°C. After 48 h, growth was assessed by a color change from purple to yellow. This color indicator was also used to study the growth of the strain at different temperatures (4, 10, 30, 35, 37, 45, or 60°C).

### Acid and bile tolerance

Determination of acid tolerance of the MZF16 strain was tested according to the protocol of Conway et al. (1987). One milliliter of overnight cultures was harvested by centrifugation (8,000 rpm, 10 min, 4°C). The cell pellets were washed three times with Phosphate Buffer Saline (PBS) and then resuspended to ~10^7^ bacteria/mL in the MRS broth pH 3.0. Aliquots of 0.1 mL were taken at 0 h and after 1, 2, and 3 h incubation at 37°C. Tolerant bacteria were assessed in terms of viable colony counts and enumerated after 24 h incubation at 37°C, by the pour plate method of all samples using decimal serial dilutions prepared in 0.1% peptone water. A sample with pH 7.0 was used as a control. Survival bacteria were expressed as log values of Colony-Forming Units per milliliter (CFU/mL).

Bile tolerance of the strain was determined by measuring growth on MRS broth with 0.3% bile salts and the method of Tambekar et al. (2009) was used with slight modifications. In brief, MRS broth having 0 and 0.3% of Ox-bile (Sigma-Aldrich) was prepared and sterilized. The MZF16 strain was grown overnight in MRS broth, it was subsequently inoculated (1% v/v) into MRS broth containing 0.3% (w/v) Ox-bile (Sigma-Aldrich). The viable colony counts were determined after 0, 1, 2, and 3 h of exposure to bile salts. Samples without addition of bile salts served as controls. After incubating for 24 h at 37°C, tolerance toward bile salts were estimated using viable counting numbers (CFU/mL).

### Autoaggregation

The assay was performed according to the protocol of Collado et al. (2008) with some minor modifications. Overnight grown *Pediococcus* at 37°C in MRS broth was harvested by centrifugation (8,000 rpm, 10 min). The cell pellet was washed twice with PBS and resuspended in the same buffer to ~10^8^ bacteria/mL. Four milliliters of the cell suspension were vortexed for 20 s and then stood at room temperature. Autoaggregation was determined at 0 and 24 h after immobilized incubation, by pipetting the upper suspension and measuring the absorbance (A) at 600 nm (A_600_). The percent difference between the initial and final absorbance gives an index of cellular autoaggregation as follows:

Autoaggregation (%) = [1 – (A_Time_/A_0_) × 100] where A_Time_ refers to the absorbance of the suspension at 24 h and A_0_, the absorbance at time 0.

The autoaggregation capacity of the strain was also evaluated by scanning electron microscopy (SEM) as previously described (Biaggini et al., 2017).

### Bacterial hydrophobicity

Ability of the microbes to adhere to hydrocarbons is a measure of their adherence to the epithelial cells in the gut also known as cell surface hydrophobicity (CSH). The CSH of MZF16 strain was determined by using the BATH (bacterial adherence to hydrocarbons) test as described by Rosenberg et al. (1980). Overnight bacterial culture was harvested by centrifugation (8,000 rpm, 10 min), washed twice with PBS and resuspended in the same buffer at ~10^8^ bacteria/mL. The cell suspension (3 mL) was vortexed with xylene, ethyl acetate or n-hexadecane (1 mL) for 2 min. The phases were allowed to separate for 20 min at room temperature leading to an obvious rise of the hydrocarbon layer. The lower aqueous phase was warily transferred to a new tube and the absorbance at 600 nm was determined.

Hydrophobicity (%) was calculated using the following equation:

H% = [(A_0_ – A)/A_0_] × 100, where A_0_ and A are absorbance values measured before and after solvent extraction.

### Caco-2/TC7 culture

The human colon adenocarcinoma cell line Caco-2/TC7 (non-mucus secreting) was used between passages 40–60. Caco-2/TC7 cells were cultured in Dulbecco's Modified Eagle's Medium (DMEM, Invitrogen, France), supplemented with 15% heat-inactivated (30 min, 56°C) fetal calf serum (FCS), and 100 U/mL each of penicillin and streptomycin. Cells were grown at 37°C under the atmosphere of 5% CO_2_ and 95% air with regularly medium change until a confluent monolayer was obtained.

### *In vitro* adhesion assay

The quantitative binding of *P. pentosaceus* MZF16 culture was investigated on Caco-2/TC7 cell line by a main approach i.e., enumeration by plating on MRS. A MZF16 culture was grown overnight, centrifuged at 8,000 rpm for 10 min at 4°C, and the pellets were washed twice in PBS and resuspended in cell culture medium, without serum and antibiotic, at a concentration of 10^8^ bacteria/mL, and then applied on confluent Caco-2/TC7 monolayers. After 3 h of incubation at 37°C, in 5% CO_2_-95% air atmosphere, monolayers were gently washed three times with sterile pre-warmed PBS to remove the bacterial suspensions and non-adherent bacteria and lysed by incubation for 15 min with 0.1% (v/v) Triton X-100 (Sigma, Aldrich) solution to detach the adherent bacteria. The lysates were then diluted, and the bacteria were enumerated on MRS agar medium. The adhesion properties were recorded as the adhesion percentage of the applied bacteria.

### Haemolytic activity and cytotoxicity assay

*P. pentosaceus* MZF16 was tested for haemolytic activity by streaking on Tryptic Soy Agar (TSA) with 5% (v/v) sheep red blood cells. Plates were incubated at 37°C for 24 h and then determined for alpha, beta or gamma-haemolysis as described by Borges et al. (2013).

Simultaneously, the cytotoxicity of MZF16 was determined using an enzymatic assay (Cytotox 96 Promega, France) which measures lactate dehydrogenase (LDH) released from the cytosol of damaged Caco-2/TC7 cells into the supernatant. LDH is a stable cytosolic enzyme present in many different cell types, particularly in eukaryotic cells, playing the role of an indicator of necrotic cell death when released. After overnight incubation with the *P. pentosaceus* MZF16 strain (10^8^ bacteria/mL), the supernatants from confluent Caco-2/TC7 monolayers grown on 24-well tissue culture plates were collected and the concentration of the LDH was quantified. Caco-2/TC7 cells exposed to Triton X100 (0.9%) were used as a positive control of maximal LDH release (100% lysis) as specified by the manufacturer's recommendations. The background level (0% LDH release) was determined with serum free culture medium. To complete the cytotoxicity assay, the integrity of Caco-2/TC7 monolayers was also estimated by observation with a photonic microscope (X400).

### Quantification of IL-8

After overnight incubation with 10^8^ bacteria/mL of *P. pentosaceus* MZF16, the levels of interleukin-8 (IL-8) produced by Caco-2/TC7 cells and released in the culture supernatant were quantified using the Human IL-8/CXCL8 Quantikine ELISA kit (R&D systems, France) according to the manufacturer's protocol.

### Transepithelial electrical resistance (TEER)

The transepithelial electrical resistance of differentiated Caco-2/TC7 cells was monitored during 16 h using the Millicell Electrical Resistance System (Millipore Corp, Bedford, MA). To assess the potential effect of *P. pentosaceus* MZF16 strain on the epithelial barrier integrity, this bacterium was incubated at 10^8^ bacteria/mL on the Caco-2/TC7 cell monolayers. Control monolayers were not exposed to the potential probiotic.

### Enzyme profile of *P. pentosaceus* MZF16

API ZYM system (BioMerieux, France) was used to evaluate the enzymatic activities of the strain *P. pentosaceus* MZF16 (Humble et al., 1977). The API strips were inoculated with an overnight bacterial culture grown in MRS broth, subsequently incubated at 37°C for 4 h. The appraisal of the enzymatic activity was carried out according to the intensity of coloration.

### Antibacterial activity of *P. pentosaceus* MZF16

Antibacterial potential of probiotics is thought to be a significant functional criterion for competitively excluding or inhibiting the activities of pathogenic intestinal microflora via production of antimicrobial compounds such as organic acids, hydrogen peroxide or bacteriocins. Antagonistic activities of the MZF16 strain was recorded against *L. innocua* HBP13, *L. monocytogenes* CIP 55143, *Enterococcus faecalis* ATCC 29212, and *Pseudomonas aeruginosa* PAO1 following the agar well diffusion assay protocol (Kim and Rajagopal, 2001). In brief, antimicrobial activity of *P. pentosaceus* MZF16 was assessed using cell-free culture supernatants (CFCS). TSA agar was seeded with the indicator strains mentioned above and solidified onto petri dishes, then 6 mm wells were formed. One hundred microliters of filtered sterilized CFS of MZF16 were then added to each well, followed by 24 h incubation at 37°C. The diameter of zone of inhibition around each well was measured and a clear zone of 1 mm or more was considered positive inhibition. The experiment was performed in triplicate.

### Characterization of the bacteriocin

Primers P1 (5′-AAAATATCTAACTAATACTTG-3′) and P2 (5′-TAAAAAGATATTTGA CCAAAA-3′) and conditions described by Rodriguez et al. (1997) were used to search for pediocin. Amplification conditions were as follow: 2 min at 95°C, 30 cycles of 1 min at 94°C, 35 s at 45°C, and 1 min at 72°C, and final elongation step 7 min at 72°C. The amplified fragments were purified via PureLink PCR purification Kit (Thermo Scientific, France) according to the manufacturer's recommendations, visualized in 1% agarose gels and sequenced by GENEWIZ. Similarity searches were conducted with the BLAST program from NCBI.

### Detection of virulence factors

*P. pentosaceus* MZF16 was subjected to the detection of virulence determinants, via PCR amplification, in order to identify its virulence activity disclosing the presence of genes encoding for aggregation protein (*agg*), gelatinase (*gelE*), and enterococcal surface protein (*esp*) (Eaton and Gasson, 2001). PCR for virulence determinant genes was performed using PCR protocols of (Mannu et al., 2003).

### Antibiotic susceptibility testing

The antibiotic susceptibility of *P. pentosaceus* MZF16 was assessed according to the technical guidelines of the European Food Safety Authority (EFSA, 2012). Minimal inhibitory concentrations (MICs) of eight antibiotics, ampicillin, vancomycin, gentamycin, erythromycin, ofloxacin, streptomycin, tetracycline, and chloramphenicol on *P. pentosaceus* MZF16 were determined by the broth microdilution method. Briefly, 100 μl of *P. pentosaceus* strain (10^5^ bacteria/well final concentration) were inoculated into the wells of a 96 well-plate containing 100 μl of each antibiotic in serial two-fold dilutions from 1,024 to 0.125 μg/mL, then were incubated at 37°C for 24 h without shaking. MICs were defined as the lowest concentration of antibiotics that inhibited the visible growth of the bacterium.

### Statistical analysis

The data collected from each experiment were expressed as a mean ± standard error (SE) calculated over three independent experiments performed in triplicate. When necessary, analysis of statistical significance was performed using GraphPad Prism software and Student's *t*-test.

## Results and discussion

### Identification of *P. pentosaceus* MZF16 by MALDI-TOF MS and 16S rDNA sequencing

A collection of fourty samples of “Dried Ossban,” obtained from homemade production of fermented meat were examined to isolate potential probiotics from the lactic acid microbiota. The MZF16 strain was selected for identification by MALDI-TOF MS Biotyper and 16S rDNA sequencing. The analysis by MALDI Biotyper classify this isolate as *P. pentosaceus* with a score value >2 which is the set threshold for match at the species level (Sogawa et al., 2011). 16s rDNA sequencing using the universal bacterial gene primers 63F/1492R and BLAST analysis in the GenBank database confirmed this identification.

### Hierarchical cluster analysis

**Figure 1:**
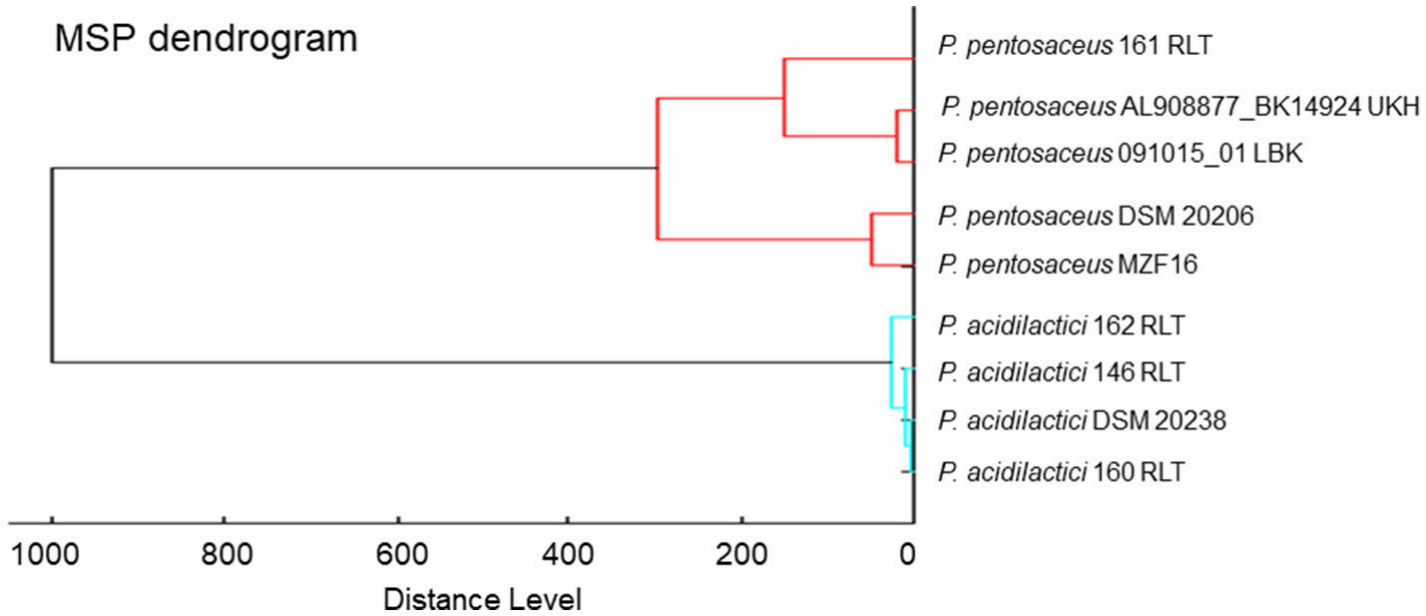
MALDI-TOF MS-based phylogenetic tree of *P. pentosaceus* MZF16 isolated from “Dried Ossban” compared to the Biotyper library.

MALDI-TOF MS hierarchical cluster analysis was used to determine the relatedness between the MS spectrum of *P. pentosaceus* MZF16 and the MS spectra available in the Biotyper library. For this, spectra were merged, and the merging patterns of spectra were then represented as dendrograms or tree structures (Figure 1). The distance of branches on the dendrograms relates directly to the similarity of spectra and, hence, of the bacteria. The results show that *P. pentosaceus* MZF16 is closely related to the reference strain *P. pentosaceus* DSM 20206, a food originated strain, which was historically named *Streptococcus citrovorus* (Hammer, 1920).

### Morphological and physiological characteristics of the MZF16 strain

The *P. pentosaceus* MZF16 strain is a gram-positive, catalase-negative coccus. Colonies of this bacteria appeared on agar as milky white, circular, concave, mucoid and glistening (Table 1). Growth at various salinity conditions and temperatures showed that MZF16 strain displayed the potential to grow in the presence of 2% NaCl and within a temperature range oscillating from 30 to 45°C.

**Table 1:** Morphological, biochemical, and physiological characteristics of the isolate MZF16.

**Characteristics**	**MZF16 (*Pediococcus pentosaceus*)**
Gram stain reaction	Gram-positive cocci
**COLONY MORPHOLOGY**	
Color	Milky white
Shape	Circular
Elevation	Concave
Density	Mucoid and glistening
**BIOCHEMICAL CHARACTERISTICS**	
Catalase activity	−
**PHYSIOLOGICAL CHARACTERISTICS AND GROWTH IN MRS MEDIUM**	
**CONDITIONS**	
With 0.5% NaCl	+
With 2% NaCl	+
With 4% NaCl	−
With 6.5% NaCl	−
With 9.5% NaCl	−
At 4°C	−
At 10°C	−
At 30°C	+
At 35°C	+
At 37°C	+
At 45°C	+
At 60°C	−

*Positive results (+), negative results (−)*.

### Acid and bile tolerance

A critical step toward the selection of probiotic strains is to appraise the strain behavior under conditions that mimic to the GI tract. According to Anandharaj et al. (2015), potential probiotic bacteria must have the ability to survive under harsh conditions including low acidity (i.e., gastric conditions) and high bile salts concentration (i.e., in the small intestine), to successfully pass through the gut, supplying health benefits to the host.

The results of acid and bile tolerance of MZF16 are presented in Table 2. When the pH of the MRS broth was decreased to pH 3, a marginal reduction of the cell viability of MZF16 was observed. The initial counts of MZF16 were reduced from 7.12 to 6.92, 6.86, and 6.81 CFU/mL after 1, 2, and 3 h, respectively. Other authors found similar survival ability of *Pediococcus* strains in harsh conditions, like Abbasiliasi et al. (2012) who showed that 97% of the population of *P. acidilactici* Kp10 survived after 3 h incubation at pH 3, or Barbosa et al. (2015) with the *P. acidilactici* HA-6111 strain, and Ilavenil et al. (2016) for *P. pentosaceus* KCC-23. Table 2 also shows that MZF16 survived after 3 h exposure to 0.3% bile salts, reflecting the critical concentration used for the selection of resistant strains (Gilliland et al., 1984). Indeed, compared to the control, only a marginal decrease was observed in bile salts supplemented MRS broth, this is due to the toxic aspect of these salts in nature (Corzo and Gilliland, 1999). The present findings coincide with previous reports that showed high survival rates of *Pediococcus* in the presence of 0.3% (w/v) of bile salts (Abbasiliasi et al., 2012; Barbosa et al., 2015; Ilavenil et al., 2016). Even more, in a study performed by Noohi et al. (2016), *P. acidilactici* and *P. pentosaceus* strains showed tolerance in 0.4% bile salts reflecting a high ability of survival and proliferation in intestinal conditions.

**Table 2:** Cell viability of *P. pentosaceus* MZF16 in various conditions of pH and concentrations of bile salts.

**Time of exposure (h)**	**Cell viability (log**_**10**_ **CFU/mL)**			

	**pH conditions**		**Concentration of bile salts (%)**	
	**Control**	**pH 3**	**Control**	**0.3**
0	6.96	7.12	6.89	6.87
1	7.01	6.92	6.97	6.81
2	7.14	6.86	7.06	6.74
3	7.26	6.81	7.18	6.69

### Autoaggregation and hydrophobicity

**Figure 2:**
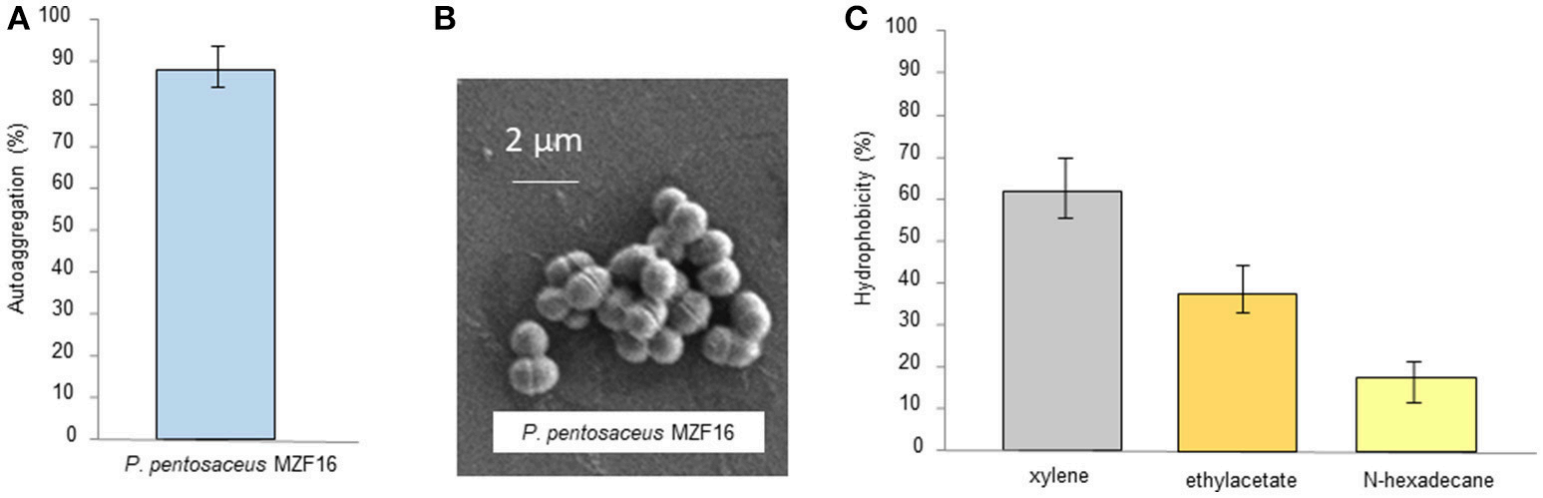
**(A)** Autoaggregation of *P. pentosaceus* MZF16, **(B)** Scanning Electron Microscopy (SEM) micrograph showing autoaggregation of MZF16, and **(C)** Assessment of cell-surface hydrophobicity of MZF16.

Autoaggregation signifies the clumping of bacterial cells from the same strain. It has been found a strong correlation between autoaggregation of a probiotic strain and its adhesion capacity to the intestinal epithelial cells, indicating a central prerequisite for successful colonization and improvement of the persistence in the gastrointestinal tract. According to Wang et al. (2010), valuable autoaggregation ability must be superior to 40%, and any strains with the ability < 10% are considered to have weak autoaggregation potential. The MZF16 strain exhibited a high level of autoaggregating ability, about 88% (Figure 2A). This capacity of autoaggregation is also apparent when the bacteria are observed by SEM (Figure 2B).

Similar results have been found in the study of Vidhyasagar and Jeevaratnam (2012) in which *P. pentosaceus* VJ41 strain exhibited maximum aggregation of 89% revealing the clumping of cells. The aggregation potential of MZF16 is quite higher than the results presented in the work of Lee et al. (2014) where the three studied *Pediococcus* strains showed aggregative abilities between 65.2 and 69.1%, than the study of Ilavenil et al. (2016) with the *P. pentosaceus* KCC-23 strain (67.3% autoaggregation) and the research of Abbasiliasi et al. (2017) in which *P. acidilactici* Kp10 (35.2% autoaggregation). Thus, it is difficult to articulate a standard rate for a high autoaggregation value for *Pediococcus* strains. Nevertheless, the value reported herein can be considered relatively reliable.

Hydrophobicity is one of cell surface physicochemical features that able to affect autoaggregation and adhesion of bacteria to various types of surfaces (Balakrishna, 2013). It has been revealed the strong connection between autoaggregation of LAB and their adhesive potential (Collado et al., 2008). The affinity of the MZF16 strain, toward various solvents such as xylene, ethyl acetate and n-hexadecane, was examined. It exhibited values of 61, 38, and 18% of hydrophobicity ability, respectively (Figure 2C). Our findings are higher than those reported by Lee et al. (2014) in which hydrophobicity with xylene and n-hexadecane of three *P. pentosaceus* strains did not exceed 34 and 4% correspondingly. Our value is higher than others cited in various studies including those of Ilavenil et al. (2016) where *P. pentosaceus* KCC-23, an isolated strain from Italian ryegrass, exhibited 32.9% of hydrophobicity to xylene. An investigation conducted by Puniya et al. (2016), in which, a *Lactobacillus* strain LHI7, exhibited values of 44.9 and 86.8% of adhesion to xylene and n-hexadecane. Results shown in this study are much higher than found in the research performed by Abbasiliasi et al. (2017), where *P. acidilactici* Kp10 showed 46.9, 5.7, and 14.5% of affinity to xylene, ethyl acetate and n-hexadecane, respectively. High hydrophobicity is firmly linked to the glycoproteins on the bacterial surface while low hydrophobicity is associated to the presence of polysaccharides on the bacterial surface (Bellon-Fontaine et al., 1996). It is important to know that the hydrophobic potential may vary between different organisms, strains, and is also influenced by age and surface chemistry of bacterial strains along with the medium components (García-Cayuela et al., 2014).

### Adhesive potential of *P. pentosaceus* MZF16 strain

**Figure 3:**
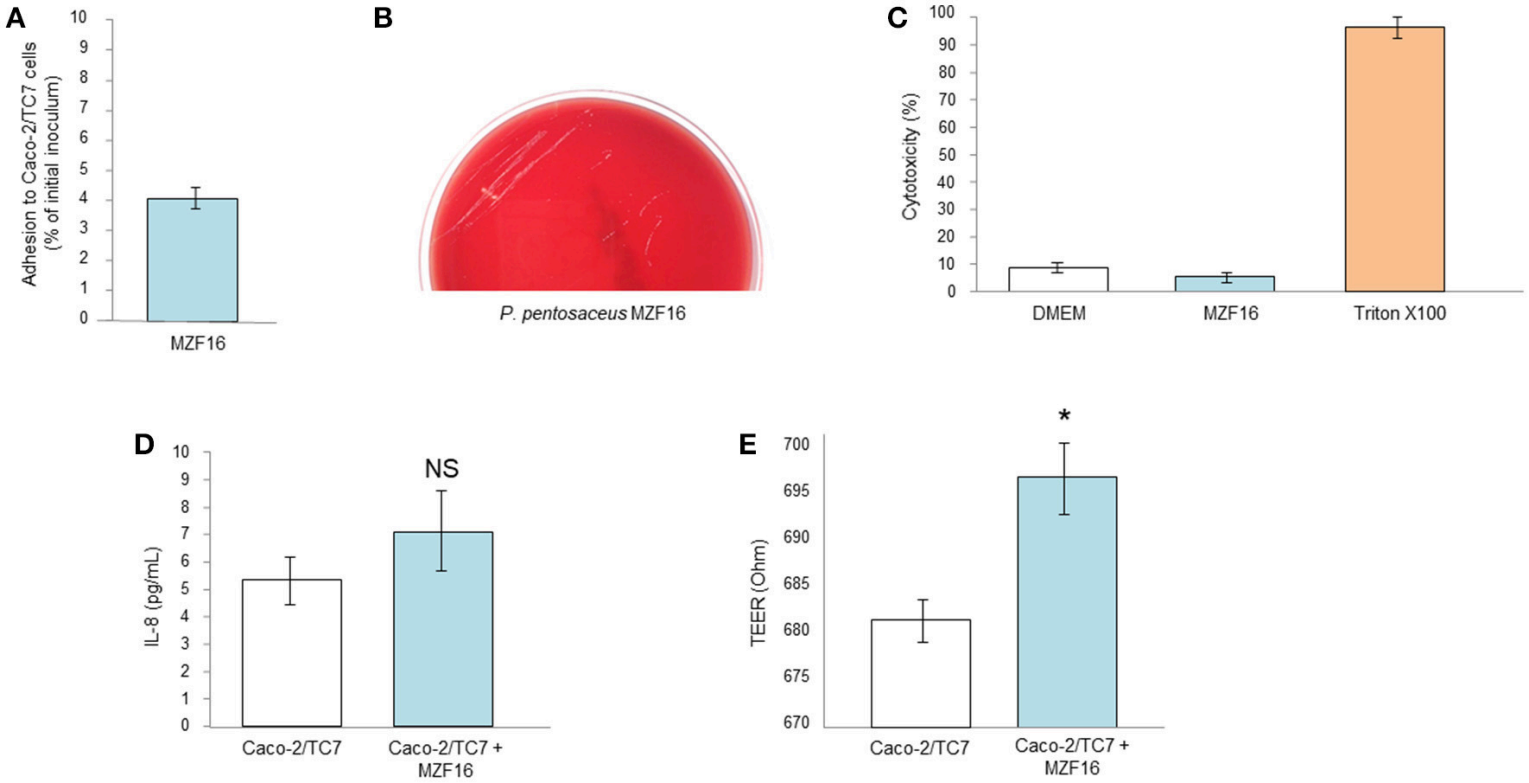
Interactions of *P. pentosaceus* MZF16 with eukaryotic cells. **(A)** Adhesion to Caco-2/TC7 cells, **(B)** Haemolytic activity, **(C)** Cytotoxicity toward Caco-2/TC7 cells, **(D)** Secretion of IL-8 by Caco-2/TC7 cells after exposition to MZF16, **(E)** TEER of Caco-2/TC7 cells exposed to MZF16. NS, not significant, *P < 0.05.

According to Blum et al. (1999), adhesion to intestinal mucosal cells, such as Caco-2 or HT 29, is considered a salient precondition screening approach for appraisal the adhesive properties of new probiotic strains. Adhesion capacity of the MZF16 strain was assessed using Caco-2/TC7 cells, originated from human epithelial colorectal adenocarcinoma cells. *P. pentosaceus* MZF16 showed weak adherence ability to Caco-2/TC7 cells, about 4% (Figure 3A). Our findings are in discordance with the study conducted by Oh and Jung (2015) who showed more than 65% adhesion levels with *P. pentosaceus* SW01 and *P. acidilactici* SW05 but the experiment has been conducted on the mucus secreting HT-29 cells, so the mucus may have promoted adhesion of the bacteria. Another investigation performed by Vidhyasagar and Jeevaratnam (2012), found that two *P. pentosaceus* strains VJ13 and VJ49 exhibited adhesive potential about 17% which is not quite different from our value of 4%. According to Del Re et al. (2000), the cell surface attributes may be used for preliminary screening to identify potentially adherent strains. However, the interpretation of the results should be taken with caution, because this adherence does not necessarily reflect *in vivo* cell adhesion (Bautista-Gallego et al., 2013).

### Haemolysis, cytotoxicity, and inflammatory potential

Haemolysis represents the ability of bacteria to lyse the blood cells. Haemolysin plays an important role in virulence increasing, as a result, the possibility of infection (Morandi et al., 2006). Haemolytic activity was studied on TSA containing 5% (v/v) sheep red blood cells (Figure 3B). At the end of incubation (24 h at 37°C), only small white colonies were visible without zones of clearing around the colonies. This can be interpreted as absence of haemolysis activity of *P. pentosaceus* MZF16 (gamma-haemolysis). The same result has been previously obtained with *P. pentosaceus* SB83, a vaginal probiotic (Borges et al., 2013), with *P. pentosaceus* MP1 (Ryu and Chang, 2013) and with *P. pentosaceus* ID9 (Narayanan et al., 2017).

Concomitantly, *P. pentosaceus* MZF16 was applied on Caco-2/TC7 monolayers for 16 h and the cytotoxicity was assessed by measurement of LDH (Lactate Dehydrogenase) release and microscopic observation. The results showed a level of cells mortality similar with MZF16 than the negative control (DMEM) representing natural mortality (Figure 3C). These findings are in line with the study of Er et al. (2015), in which the cytotoxicity of three LAB (*P. pentosaceus, Lactobacillus plantarum*, and *Weissella confusa*) has been studied on Caco-2 cells.

Human intestinal cell lines have been shown to constitutively produce the interleukin 8 (IL-8). IL-8 is a member of the C-X-C chemokine family and plays an essential role in the recruitment and activation of neutrophils, thereby initiating the inflammatory response. In this study, we examined the level of IL-8 released in cell-free supernatant of Caco-2/TC7 cells incubated with *P. pentosaceus* MZF16. As shown in Figure 3D, MZF16 did not affect IL-8 production by Caco-2/TC7 cells compared to untreated cells, suggesting that MZF16 does not induce an inflammatory response.

### Transepithelial electrical resistance measurement

Under culture conditions on inserts, Caco-2/TC7 cells develop morphological and functional traits of enterocytes, including intercellular tight junctions. Thus, transepithelial electrical resistance may ensure the measurement of the integrity of the tight junctions. After 16 h of incubation, MZF16 showed a slight increase in the TEER measurement reflecting one of the important criteria of probiotic (Figure 3E). Our results are in harmony with other investigations of different characterized probiotics (*Bifidobacterium infantis, Lactobacillus acidophilus, Lactobacillus rhamnosus, Escherichia coli* Nissle 1917, *Streptococcus thermophilus*, the probiotic complex VSL#3) on several cell lines (T84, HT29/cl.19A, and Caco-2) (Resta-Lenert and Barrett, 2003; Sherman et al., 2005; Ewaschuk et al., 2008). The application of probiotics on intestinal epithelial cells may generally lead to improvement of TEER values (Klingspor et al., 2015).

### Enzyme activities

Several enzyme activities included in carbohydrate, protein, lipid, and phosphate metabolism were examined by using API-ZYM kit. The MZF16 strain demonstrated strong peptidases activities (Leucine arylamidase and Valine arylamidase). It also showed N-acetyl-b-glucosaminidase, Acid phosphatase, and β-Glucosidase activities at high levels (Table 3). Additionally, moderate levels of activity of Cystine arylamidase, Naphthol-AS-BI-phosphohydrolase have been observed. β-Glucosidase (β-Glu) plays a central role in the bioconversion of glycosides into aglycones, which are more easily absorbed in human intestines and thus considered biologically more active forms than corresponding glycosides (Chun et al., 2007). In our present study, *P. pentosaceus* MZF16 produced different types of enzymes encompassing glycolytic enzymes. This could be useful in the fermented food products related industries.

**Table 3:** Enzyme activities of *P. pentosaceus* MZF16 determined by API-ZYM kit.

**Enzyme**	**Substrate**	***P. pentosaceus* MZF16**
Control	−	−
Alkaline phosphatase	2-naphthyl phosphate	−
Esterase	2-naphthyl butyrate	−
Esterase Lipase	2-naphthyl caprylate	−
Lipase	2-naphthyl myristate	−
Leucine arylamidase	L-leucyl-2-naphthylamide	++++
Valine arylamidase	L-valyl-2-naphthylamide	++++
Cystine arylamidase	L-cystyl-2-naphthylamide	++
Trypsin	N-benzoyl-DL-arginine-2 naphthylamide	−
α-chymotrypsin	N-glutaryl-phenylanine-2-naphthylamide	−
Acid phosphatase	2-naphthyl phosphate	++++
Naphtol-AS-BI- phosphohydrolase	Naphtol-AS-BI-phosphate	++
α-galactosidase	6-Br-2-naphthyl-α-D-galactopyranoside	−
β-galactosidase	2-naphthyl-β-D-galactopyranoside	−
β-glucuronidase	Naphtol-AS-BI-β-D-glucuronide	−
α-glucosidase	2-naphthyl-α-D-glucopyranoside	−
β-glucosidase	6-Br-2-naphthyl-β-D-glucopyranoside	++++
N-acetyl-β- glucosaminidase	1-naphthyl-N-acetyl-β-D-glucosaminide	++++
α-mannosidase	6-Br-2-naphthyl-α-D-mannopyranoside	−
α-fucosidase	2-naphthyl-α-L-fucopyranoside	−

*(−), negative (colorless or very pale yellow); (+), positive (violet or orange or blue)*.

### Antimicrobial activity

Antagonistic activity of LAB against spoilage and pathogenic bacteria is regarded as a probiotic trait, in order to maintain the balance of the gut microflora and to keep the gut rid of pathogens. The microbial antagonism of probiotics comprises the production of non-specific antimicrobial substances including short-chain fatty acids (SCFA), hydrogen peroxide and low-molecular-weight proteins so called bacteriocins and bacteriocin-like inhibitory substances (BLIS) which are among the major reported compounds with antimicrobial potential (Cruz-Guerrero et al., 2014). LAB are well-known to produce a wide range of antimicrobial compounds of which bacteriocin is widely studied (Schnurer and Magnusson, 2005). Numerous investigations evidenced the production of bacteriocins by Pediococci with wide spectrum of activity (Rodríguez et al., 2002; Jamuna and Jeevaratnam, 2004; Papagianni and Anastasiadou, 2009).

**Figure 4:**
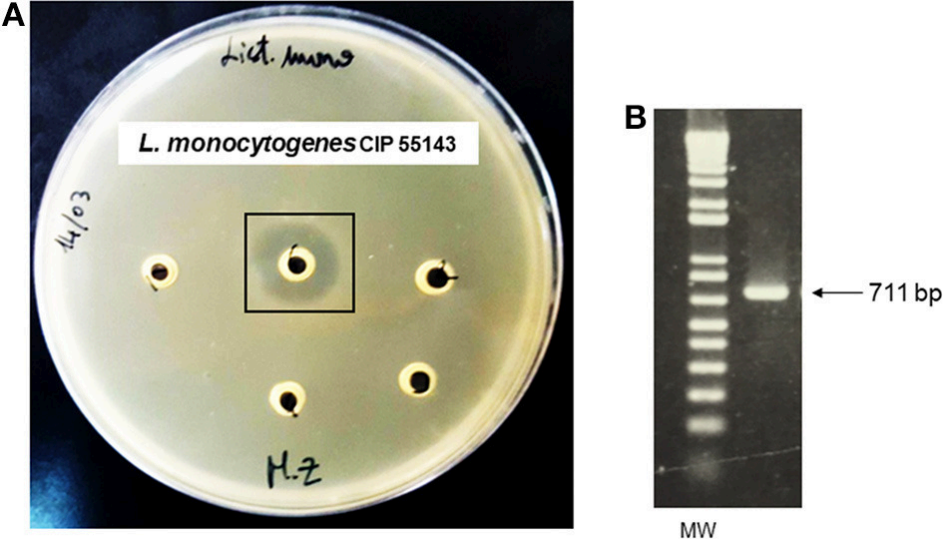
**(A)** Antibacterial activity of *P. pentosaceus* MZF16 against *Listeria monocytogenes* CIP 55143 as demonstrated by the inhibition zone, **(B)** Detection of pediocin gene in MZF16.

Antagonistic activity exhibited by *P. pentosaceus* MZF16 was determined against spoilage and pathogenic bacteria viz. *L. monocytogenes* CIP 55143, *L. innocua* HBP13, *E. faecalis* ATCC 29212, and *P. aeruginosa* PAO1. The results showed that *P. pentosaceus* MZF16 is strongly active against *L. monocytogenes* CIP 55143 which is an important food-borne pathogen (Table 4, Figure 4A). This observation could infer that *P. pentosaceus* MZF16 has the potential to be used as a probiotic microorganism, in order to conquer some serious challenges facing the food industry and regulatory agencies. Additionally, MZF16 showed resistance to its own bacteriocin as indicated by the absence of activity around the well (Table 4). All bacteriocin-producing LAB have a self-immunity ability from the adverse effect of their own bacteriocins via the production of an immune protein directly associated with the C-terminal domain of the bacteriocin (Bharti et al., 2015).

**Table 4:** Inhibitory spectrum of the cell-free culture supernatant (CFCS) of *P. pentosaceus* MZF16 as measured by agar well diffusion method.

**Indicator strains**	Inhibition zone (mm)^*^of MZF16			

	**Non-treated CFCS**	**Neutralized at pH 6.5**	**Proteinase K**	**Heat treatment**
*L. innocua* HBP13	+++	++	−	++
*L. monocytogenes* CIP 55143	+++	++	−	++
*E. faecalis* ATCC 29212	+++	+++	−	++
*P. aeruginosa* PAO1	++	++	−	+
*P. pentosaceus* MZF16	−	−	−	−

* *, No inhibition zone; + < 3 mm, ++ 3–6 mm, +++ radius inhibit zone > 6 mm*.

In this study, the MZF16 strain produces an antimicrobial substance active at pH 6.5. The activity was lost after treatment with proteinase K (protease) which proves that the antimicrobial substance is bacteriocin in nature (Moraes et al., 2010).

### Characterization of the bacteriocin and immunity protein

**Figure 5:**
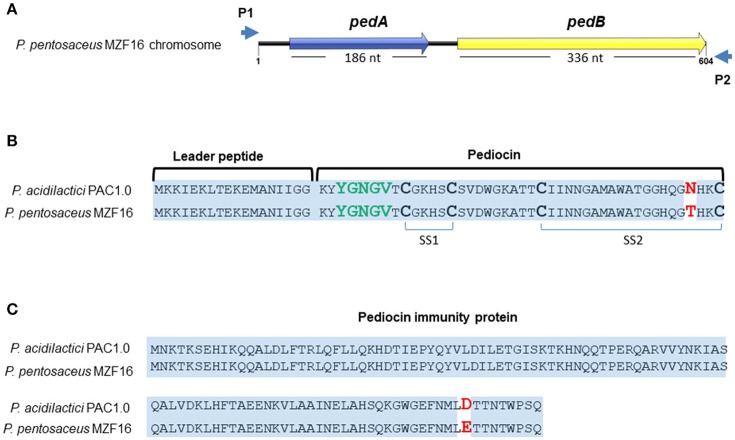
Sequences of pediocin MZF16 **(A,B)** and its immunity protein **(A,C)**. The amino acid sequences of pediocin MZF16 and immunity protein of *P. pentosaceus* MZF16 have been compared with *P. acidilactici* PAC1.0 using BlastX.

The bacteriocin produced by *P. pentosaceus* MZF16 has been investigated by PCR amplification and sequencing. For this, primers P1 and P2 were used to amplify a 711-bp DNA fragment (Figure 4B) and to sequence each DNA strand (Figure 5A) (from 91 bp upstream of pedA to 33 bp downstream of the translational start of pedC) (Mathys et al., 2007) designed from the pediocin PA-1 (ACH) operon of *P. acidilactici* PAC1.0 (Marugg et al., 1992) that allow to amplify both the PedA structural gene of the bacteriocin and PedB encoding the immunity protein.

These two ORFs were translated to amino acid (AA) sequences and compared to *P. acidilactici* PAC1.0 bacteriocin and immunity protein using BlastX and Bactibase, a database dedicated to bacteriocins (Figures 5B,C). The results showed a high sequence identity of these peptides between the two *Pediococci* (Figure 5B). The pedA structural gene (Figure S1) encodes the putative pre-pediocin composed of 62 residues (18 for the leader peptide and 44 for the mature pediocin). The putative mature pediocin MZF16 contains the consensus sequence YYGNGVXCXXXXCXVXXXXA, with the YGNGV motif responsible for the anti-*Listeria* activity, and the two-disulfide bonds, characteristics of class IIa bacteriocins, and includes a mismatch at the C-terminus of the peptide, compared to the known pediocins previously characterized, produced by *P. acidilactici* or *P. pentosaceus* strains. Hence, a threonine residue was present at position 41 (T41) in pediocin MZF16 instead of an asparagine residue (N41) for pediocin PA-1 (AcH) of *P. acidilactici* PAC1.0 and other pediocins (Figure 5B). The same substitution has been found before in coagulin A, the pediocin-like bacteriocin produced by *Bacillus coagulans* (Le Marrec et al., 2000) which is 100% identical to the pediocin MZF16. This finding is interesting, as to our knowledge, this represents the first report of such modification in the sequence of a pediocin in a *Pediococcus* strain.

Anti-*Listeria* class IIa bacteriocins are important antimicrobial peptides for use as food preservatives as their low molecular weight allow them to be stable to heat process.

The putative immunity protein of MZF16 also showed high similarity with *P. acidilactici* PAC1.0 (Figure 5C) with only one substitution at position 144 where a glutamic acid (E144) is present instead of an aspartic acid (D144).

**Figure 6:**
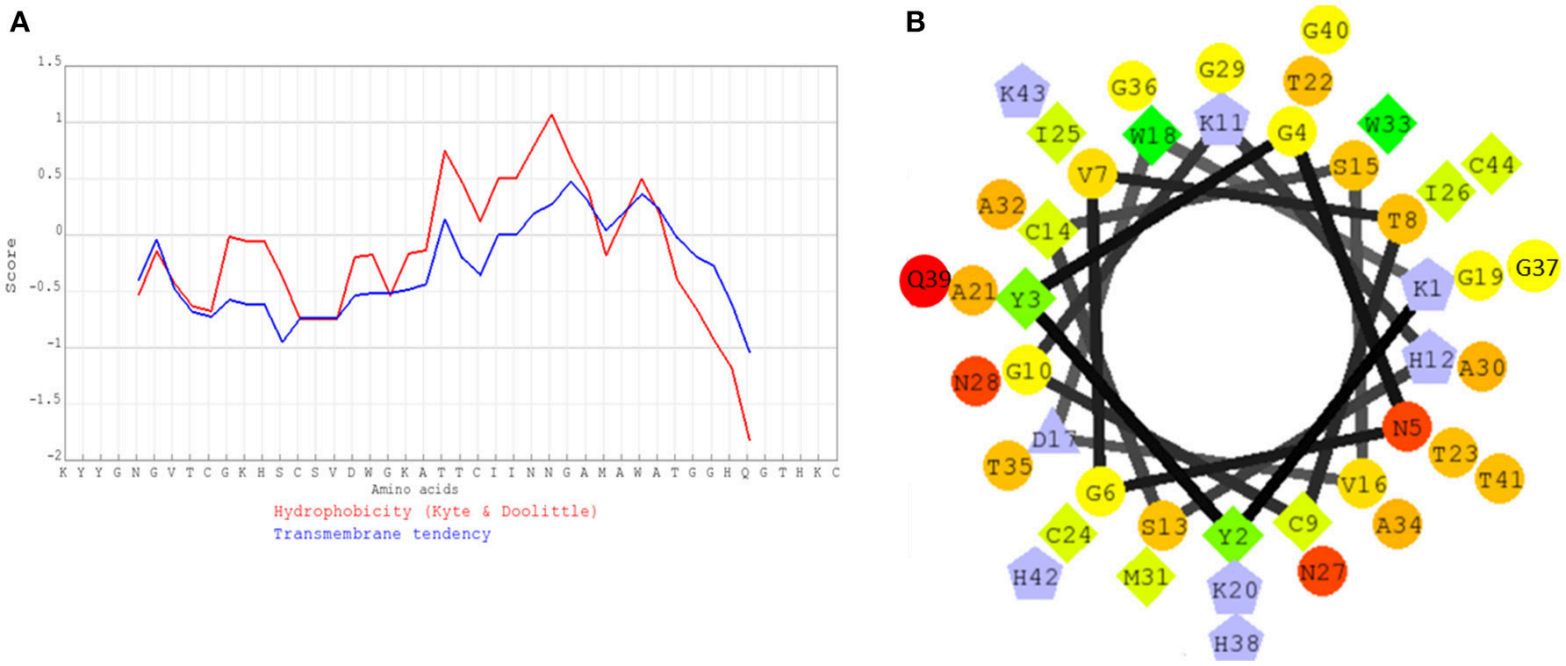
**(A)** Hydrophobicity profile of pediocin MZF16, **(B)** Helical wheel structure of pediocin MZF16.

The hydrophobicity profile (Figure 6A) and helical wheel structure (Figure 6B) of pediocin MZF16 was constructed by similarity to coagulin A (http://bactibase.hammamilab.org/BAC084). The hydrophobic properties and helical structure of class IIa bacteriocins enable them to interact with the phospholipids of the membrane and create pores in indicator strains as *Listeria* spp. (Drider et al., 2006; Ríos Colombo et al., 2017).

### Antibiotic susceptibility of the mzf16 strain

The MZF16 strain was subjected to commonly prescribed antibiotics (Table 5): a DNA replication and transcription inhibitor (Ofloxacin), two cell wall synthesis inhibitors (Ampicillin and Vancomycin) and numerous protein synthesis inhibitors (Chloramphenicol, Erythromycin, Gentamycin, Streptomycin, and Tetracycline). *P. pentosaceus* MZF16 was sensitive to five antibiotics, resistant only to gentamycin, chloramphenicol, and vancomycin. Antibiotic susceptibility results are referred with reported strains of *Pediococcus pentosaceus* CRAG3 (Shukla and Goyal, 2014), *P. pentosaceus* KID7 (Damodharan et al., 2015), *P. pentosaceus* KCC-23 (Ilavenil et al., 2016), *P. pentosaceus* ID9 (Narayanan et al., 2017), *P. pentosaceus* ST65ACC (Cavicchioli et al., 2018), *P. pentosaceus* LJR1 (Ladha and Jeevaratnam, 2018), Shi et al. (2018) with minor variations.

**Table 5:** Minimum inhibitory concentration (MIC; μg/mL) of eight antibiotics toward *P. pentosaceus* MZF16 strain.

**Class**	**Antibiotic**	**MIC (**μ**g/mL)**		

		***P. pentosaceus*** **MZF16**	**MIC breakpoint recommendation of EFSA for** ***Pediococcus***	**Antibiotic Susceptibility**
β-Lactams	Ampicillin	2	4	S
Tetracyclines	Tetracycline	8	8	S
Macrolide	Erythromycin	1	1	S
Quinolones	Ofloxacin	16	N.R	S^*^
Aminoglycoside	Streptomycin	64	64	S
	Gentamycin	32	16	R
Chloramphenicol	Chloramphenicol	8	4	R
Glycopeptides	Vancomycin	>64	N.R	R

EFSA, European Food Safety Authority; MIC, Minimal Inhibitory Concentration; N.R, Not Required by EFSA guideline; R, resistant; S, susceptible; S

* *Susceptible according the MIC breakpoints provided by Danielsen et al. (2007) for Pediococcus genus*.

Several reports have shown that LAB are generally resistant to various antibiotics (Temmerman et al., 2003; Osmanagaoglu et al., 2010; Bhakta et al., 2012; Monteagudo-Mera et al., 2012). The resistance to vancomycin presented by the MZF16 strain was inherent in the *Pediococcus* genus and is commonly reported (Toomey et al., 2010; Franz et al., 2014). This intrinsic resistance to vancomycin is due to a modified peptidoglycan precursor ending in D-Ala-D-lactate (Billot-Klein et al., 1994). According to Ammor et al. (2007), intrinsic resistance is not horizontally transferable and poses no risk in non-pathogenic bacteria. Two pilot investigations carried out by Danielsen et al. (2007) and Franz et al. (2014) exhibited that *Pediococcus* spp. is intrinsically resistant to numerous groups of antibiotics encompassing β-lactams, cephalosporins, aminoglycosides, glycopeptides, streptomycin, kanamycin, tetracyclines, and sulfa (with or without trimethoprim). In fact, antibiotic resistance as such is not a safety concern, but it becomes a serious peril when resistance is transferable (Sharma et al., 2014).

Antibiotic resistance of bacteria has increasingly become an alarming medical issue. Multi-drug resistance of pathogenic bacteria against medically important antibiotics has become a critical threat and serious challenge to go over in purpose to treat infected patients. Therefore, antibiotic susceptibility represents a crucial prerequisite for probiotics. It is relevant to know that the *P. pentosaceus* species is considered according to the EFSA to be suitable for the Qualified Presumption of Safety (QPS) approach to safety assessment (EFSA, 2007; EFSA BIOHAZ Panel, 2017). This approach requires the identity of the strain to be decisively established and evidence that the strain does not show any type of acquired resistance to antibiotics of human and veterinary importance.

### Detection of virulence determinants

*P. pentosaceus* MZF16 harbored any of the virulence genes assessed in this study. The absence of virulence determinants represents a precondition to consider a bacterium as probiotic. The virulence genes investigated herein were selected as they are associated with other related LAB, such as *Enterococcus* spp. (Barbosa et al., 2010, 2015; Johansson and Rasmussen, 2013). Our findings are in line with the study of Albano et al. (2009), in which *P. acidilactici* HA-6111-2 showed total absence of virulence determinants. For instance, several authors have reported the absence of virulence factors in *Pediococcus* spp. (Borges et al., 2013; Muñoz-Atienza et al., 2013).

## Conclusion

Pediococci are microorganisms that are still little studied. Nevertheless, interest seems to be in a non-stop rising in both fields, the scientific investigation and in the industry sector. In the last decade, various scientific researches and studies have been published, and food supplements containing *Pediococcus* spp. have invaded the nutraceutical market (Papagianni and Anastasiadou, 2009).

To sum up, *P. pentosaceus* MZF16 isolated from artisanal Tunisian meat product “Dried Ossban” demonstrated high capability to withstand harsh environmental conditions such as low pH and bile salts. It passed most of the *in vitro* properties for probiotics recommended by FAO/WHO (2002) and was sensitive to most common antibiotics. This strain exhibited also effective inhibition against food spoilage and pathogenic bacteria as *L. monocytogenes*. Its bacteriocin, the pediocin MZF16 has been found identical to coagulin A, exhibiting the originality of this study, as it represents the first report of a pediocin 100% similar to coagulin A. Its belonging to class IIa bacteriocins containing the YGNGV consensus motif, the salient criterion of pediocin-like bacteriocins with anti-listerial activity, make pediocin MZF16 so appropriate as a food preservative. Overall, *in vivo* assessment and immunomodulation tests are still required to boost the applicability of the strain and to categorically substantiate its true potential. Our findings indicate that *P. pentosaceus* MZF16 with its bacteriocin can act as a promising probiotic candidate for future use in a wide range of functional foods to prevent development of *Listeria* spp.

## Author contributions

MZ and NC carried out most of the experimental work, interpreted the data, and wrote the manuscript. EB performed the sequence analysis of pediocin MZF16. OM contributed to the PCR assays. MB helped for the identification of the MZF16 strain by MALDI-TOF MS and the construction of the dendrogram. SS realized the observation of MZF16 by scanning electronic microscopy. MoF and NC designed and co-supervised the entire project. All authors have edited, read, and approved the final version of the manuscript.

### Conflict of interest statement

The authors declare that the research was conducted in the absence of any commercial or financial relationships that could be construed as a potential conflict of interest.

## References

[B1] Abbasiliasi S. Tan J. S. Bashokouh F. Ibrahim T. A. T. Mustafa S. Vakhshiteh F. (2017). *In vitro* assessment of *Pediococcus acidilactici* Kp10 for its potential use in the food industry. BMC Microbiol. 17:121. 10.1186/s12866-017-1000-z 28535747 PMC5442676

[B2] Abbasiliasi S. Tan J. S. Ibrahim T. A. T. Ramanan R. N. Vakhshiteh F. Mustafa S. (2012). Isolation of *Pediococcus acidilactici* Kp10 with ability to secrete bacteriocin-like inhibitory substance from milk products for applications in food industry. BMC Microbiol. 12:260. 10.1186/1471-2180-12-260 23153191 PMC3571982

[B3] Albano H. Pinho C. Leite D. Barbosa J. Silva J. Carneiro L. (2009). Evaluation of a bacteriocin-producing strain of *Pediococcus acidilactici* as a biopreservative for “Alheira,” a fermented meat sausage. Food Control. 20, 764–770. 10.1016/j.foodcont.2008.09.021

[B4] Ammor M. S. Flórez A. B. Mayo B. (2007). Antibiotic resistance in nonenterococcal lactic acid bacteria and bifidobacteria. Food Microbiol. 24, 559–570. 10.1016/j.fm.2006.11.001 17418306

[B5] Anandharaj M. Sivasankari B. Santhanakaruppu R. Manimaran M. Rani R. P. Sivakumar S. (2015). Determining the probiotic potential of cholesterol-reducing *Lactobacillus* and *Weissella* strains isolated from gherkins (fermented cucumber) and south Indian fermented koozh. Res. Microbiol. 166, 428–439. 10.1016/j.resmic.2015.03.002 25839996

[B6] Balakrishna A. (2013). In vitro evaluation of adhesion and aggregation abilities of four potential probiotic strains isolated from guppy (*Poecilia reticulata*). *Braz. Arch. Biol. Technol* 56, 793–800. 10.1590/S1516-89132013000500010

[B7] Barbosa A. A. T. Mantovani H. C. Jain S. (2017). Bacteriocins from lactic acid bacteria and their potential in the preservation of fruit products. Crit. Rev. Biotechnol. 3, 1–13. 10.1080/07388551.2016.1262323 28049350

[B8] Barbosa J. Borges S. Teixeira P. (2015). *Pediococcus acidilactici* as a potential probiotic to be used in food industry. Int. J. Food Sci. Technol. 50, 1151–1157. 10.1111/ijfs.12768

[B9] Barbosa J. Gibbs P. A. Teixeira P. (2010). Virulence factors among enterococci isolated from traditional fermented meat products produced in the North of Portugal. Food Control 21, 651– 656. 10.1016/j.foodcont.2009.10.002

[B10] Bauer R. Chikindas M. L. Dicks L. M. T. (2005). Purification, partial amino acid sequence and mode of action of pediocin PD-1, a bacteriocin produced by *Pediococcus damnosus* NCFB1832. Int. J. Food Microbiol. 101, 17–27. 10.1016/j.ijfoodmicro.2004.10.040 15878403

[B11] Bautista-Gallego J. Arroyo-López F. N. Rantsiou K. Jiménez-Díaz R. Garrido-Fernández A. Cocolin L. (2013). Screening of lactic acid bacteria isolated from fermented table olives with probiotic potential. Food Res. Int. 50, 135–142. 10.1016/j.foodres.2012.10.004

[B12] Belhadj H. Harzallah D. Bouamra D. Khennouf S. Dahamna S. Ghadbane M. (2014). Phenotypic and Genotypic characterization of some lactic acid bacteria isolated from Bee Pollen: a preliminary Study. Biosci. Microbiota Food Health. 33, 11–23. 10.12938/bmfh.33.11 24936378 PMC4034326

[B13] Bellon-Fontaine M. N. Rault J. Van Oss C. J. (1996). Microbial adhesion to solvents: a novel method to determine the electron-donor/electron-acceptor or Lewis acid-base properties of microbial cells. Colloids Surface B Biointerfaces 7, 47–53. 10.1016/0927-7765(96)01272-6

[B14] Bhakta J. N. Ohnishi K. Munekage Y. Iwasaki K. Wei M. Q. (2012). Characterization of lactic acid bacteria-based probiotics as potential heavy metal sorbents. J. Appl. Microbiol. 112, 1193–1206. 10.1111/j.1365-2672.2012.05284.x 22404232

[B15] Bharti V. Mehta A. Singh S. Jain N. Ahirwal L. Mehta S. (2015). Bacteriocin: a novel approach for preservation of food. Int. J. Pharm. Pharm. Sci. 17, 20–29.

[B16] Biaggini K. Borrel V. Szunerits S. Boukherroub R. N'Diaye A. Zébr,é A.. (2017). Substance P enhances lactic acid and tyramine production in *Enterococcus faecalis* V583 and promotes its cytotoxic effect on intestinal Caco-2/TC7 cells. Gut Pathog. 9:20. 10.1186/s13099-017-0171-3 28439299 PMC5399405

[B17] Billot-Klein D. Gutmann L. Sable S. Guittet E. van Heijenoort J. (1994). Modification of peptidoglycan precursors is a common feature of the low-level vancomycin-resistant VANB-type *Enterococcus* D366 and of the naturally glycopeptide-resistant species *Lactobacillus casei, Pediococcus pentosaceus, Leuconostoc mesenteroides*, and *Enterococcus gallinarum*. J. Bacteriol. 176, 2398–2405. 10.1128/jb.176.8.2398-2405.1994 8157610 PMC205365

[B18] Blum S. Reniero R. Schiffrin E. Crittenden R. Mattila-Sandholm T. Ouwehand A. (1999). Adhesion studies for probiotics: need for validation and refinement. Trends Food Sci. Technol. 10, 405–410. 10.1016/S0924-2244(00)00028-5

[B19] Borges S. Barbosa J. Silva J. Teixeira P. (2013). Evaluation of characteristics of *Pediococcus* spp. to be used as a vaginal probiotic. J. Appl. Microbiol. 115, 527–538. 10.1111/jam.12232 23611355

[B20] Carafa I. Nardin T. Larcher R. Viola R. Tuohy K. Franciosi E. (2015). Identification and characterization of wild lactobacilli and pediococci from spontaneously fermented Mountain Cheese. Food Microbiol. 48, 123–132. 10.1016/j.fm.2014.12.003 25791000

[B21] Cavicchioli V. Q. Camargo A. C. Todorov S. D. Nero L. A. (2018). Potential control of *Listeria monocytogenes* by bacteriocinogenic *Enterococcus hirae* ST57ACC and *Pediococcus pentosaceus* ST65ACC strains isolated from artisanal cheese. Probiotics Antimicrob. Prot. [Epub ahead of print]. 10.1007/s12602-018-9449-0 30069686

[B22] Chen F. Zhu L. Qiu H. (2017). Isolation and probiotic potential of *Lactobacillus Salivarius* and *Pediococcus Pentosaceus* in specific pathogen free chickens. Braz. J. Poult. Sci. 19, 325–332. 10.1590/1806-9061-2016-0413

[B23] Chun J. Kim G. M. Lee K. W. Choi I. D. Kwon G. H. Park J. Y.. (2007). Conversion of isoflavone glucosides to aglycones in soymilk by fermentation with lactic acid bacteria. J. Food Sci. 72, M39–M44. 10.1111/j.1750-3841.2007.00276.x 17995840

[B24] Collado M. C. Meriluoto J. Salminen S. (2008). Adhesion and aggregation properties of probiotic and pathogen strains. Eur. Food Res. Technol. 226, 1065–1073. 10.1007/s00217-007-0632-x

[B25] Conway P. L. Gorbach S. L. Goldin B. R. (1987). Survival of lactic acid bacteria in the human stomach and adhesion to intestinal cells. J. Dairy Sci. 70, 1–12. 10.3168/jds.S0022-0302(87)79974-3 3106442

[B26] Corzo G. Gilliland S. E. (1999). Bile salt hydrolase activity of three strains of *Lactobacillus acidophilus*. J. Dairy Sci. 82, 472–480. 10.3168/jds.S0022-0302(99)75256-2 10194664

[B27] Cotter P. D. Hill C. Ross R. P. (2005). Bacteriocins: developing innate immunity for food. Nature. 3, 777–788. 10.1038/nrmicro1273 16205711

[B28] Cotter P. D. Ross R. P. Hill C. (2013). Bacteriocins- a viable alternative to antibiotics? Nat. Rev. Microbiol. 11, 95–105. 10.1038/nrmicro2937 23268227

[B29] Cruz-Guerrero A. Hernández-Sánchez H. Rodríguez-Serrano G. Gómez-Ruiz L. García-Garibay M. Figueroa-González I. (2014). Commercial probiotic bacteria and prebiotic carbohydrates: a fundamental study on prebiotics uptake, antimicrobials production and inhibition of pathogens. J. Sci. Food. Agric. 94, 2246–2252. 10.1002/jsfa.6549 24374769

[B30] Damodharan K. Lee Y. S. Palaniyandi S. A. Yang S. H. Suh J. W. (2015). Preliminary probiotic and technological characterization of *Pediococcus pentosaceus* strain KID7 and *in vivo* assessment of its cholesterol-lowering activity. Front. Microbiol. 6:768. 10.3389/fmicb.2015.00768 26300852 PMC4523826

[B31] Danielsen M. Simpson P. O'Connor E. Ross R. Stanton C. (2007). Susceptibility of *Pediococcus* spp. to antimicrobial agents. J. Appl. Microbiol. 102, 384–389. 10.1111/j.1365-2672.2006.03097.x 17241343

[B32] Del Re B. Sgorbati B. Miglioli M. Palenzola D. (2000). Adhesion, auto-aggregation and hydrophobicity of 13 strains of *Bifidobacterium longum*. Lett. Appl. Microbiol. 31, 438–442. 10.1046/j.1365-2672.2000.00845.x 11123552

[B33] Dobson C. M. Deneer H. Lee S. Hemmingsen S. Glaze S. Ziola B. (2002). Phylogenetic analysis of the genus *Pediococcus*, including *Pediococcus claussenii* sp. nov., a novel lactic acid bacterium isolated from beer. Int. J. Syst. Evol. Microbiol. 52, 2003–2010. 10.1099/00207713-52-6-2003 12508860

[B34] Drider D. Fimland G. Héchard Y. McMullen L. M. Prévost H. (2006). The continuing story of class IIa bacteriocins. Microbiol. Mol. Biol. Rev. 70, 564–582. 10.1128/MMBR.00016-05 16760314 PMC1489543

[B35] Dubey V. Ghosh A. R. Bishayee K. Khuda-Bukhsh A. R. (2015). Probiotic *Pediococcus pentosaceus* strain GS4 alleviates azoxymethane-induced toxicity in mice. Nutr. Res. 35, 921–929. 10.1016/j.nutres.2015.08.001 26319614

[B36] Eaton T. J. Gasson M. J. (2001). Molecular screening of *Enterococcus* virulence determinants and potential for genetic exchange between food and medical isolates. Appl. Environ. Microbiol. 67, 1628–1635. 10.1128/AEM.67.4.1628-1635.2001 11282615 PMC92779

[B37] EFSA (2007). Opinion of the Scientific Committee on a request from EFSA on the introduction of a Qualified Presumption of Safety (QPS) approach for assessment of selected microorganisms referred to EFSA. EFSA J. 5:587 10.2903/j.efsa.2007.587

[B38] EFSA (2012). Guidance on the assessment of bacterial susceptibility to antimicrobials of human and veterinary importance. EFSA J. 10, 2740–2749. 10.2903/j.efsa.2012.2740

[B39] EFSA BIOHAZ Panel (2017). Scientific Opinion on the update of the list of QPS-recommended biological agents intentionally added to food or feed as notified to EFSA. EFSA J. 15:4664 10.2903/j.efsa.2017.4664 PMC7010101 32625421

[B40] Er S. Koparal A. T. Kivan,ç M. (2015). Cytotoxic effects of various lactic acid bacteria on Caco-2 cells. Turk. J. Biol. 39, 23–30. 10.3906/biy-1402-62

[B41] Ewaschuk J. B. Diaz H. Meddings L. Diederichs B. Dmytrash A. Backer J.. (2008). Secreted bioactive factors from *Bifidobacterium infantis* enhance epithelial cell barrier function. Am. J. Physiol. Gastrointest. Liver Physiol. 295, G1025–G1034. 10.1152/ajpgi.90227.2008 18787064

[B42] FAO/WHO (2002). Guidelines for the evaluation of probiotics in food - Joint Food and Agricultural Organization of the United Nations and World Health Organization Working Group Meeting Report. London, ON.

[B43] Franz C. M. A. P. Endo A. Abriouel H. Reenen C. A. V. Gálvez A. Dicks L. M. T. (2014). The genus *Pediococcus*, in Lactic Acid Bacteria: Biodiversity and Taxonomy, eds Holzapfel W. H. Wood B. J. B. (Chichester; West Sussex, UK: Wiley Blackwell), 359–376. 10.1002/9781118655252.ch21

[B44] García-Cayuela T. Korany A. M. Bustos I. Gómez de Cadiñanos L. P. Requena T. Peláez C. (2014). Adhesion abilities of dairy *Lactobacillus plantarum* strains showing an aggregation phenotype. Food Res. Int. 57, 44–50. 10.1016/j.foodres.2014.01.010

[B45] García-Ruiz A. González de Llano D. Esteban-Fernández A. Requena T. Bartolomé B. Moreno-Arribas M. V. (2014). Assessment of probiotic properties in lactic acid bacteria isolated from wine. Food Microbiol. 44, 220–225. 10.1016/j.fm.2014.06.015 25084666

[B46] Gaspar P. Carvalho A. L. Vinga S. Santos H. Neves A. R. (2013). From physiology to systems metabolic engineering for the production of biochemicals by lactic acid bacteria. Biotechnol. Adv. 31, 764–788. 10.1016/j.biotechadv.2013.03.011 23567148

[B47] Gilliland S. E. Staley T. E. Bush I. J. (1984). Importance of bile tolerance of *Lactobacillus acidophilus* used as a dietary adjunct. J. Dairy Sci. 67, 3045–3051. 10.3168/jds.S0022-0302(84)81670-7 6442304

[B48] Gudiña E. J. Fernandes E. C. Teixeira J. A. Rodrigues L. R. (2015). Antimicrobial and antiadhesive activities of cell-bound biosurfactant from *Lactobacillus agilis* CCUG31450. RSC Adv. 5, 90960–90968. 10.1039/C5RA11659G

[B49] Halami P. M. Ramesh A. Chandrashekar A. (2005). Fermenting cucumber, a potential source for the isolation of pediocin-like bacteriocin producers. World J. Microbiol. Biotechnol. 21, 1351–1358. 10.1007/s11274-005-4858-0

[B50] Hammer B. W. (1920). Volatile acid production of *S. lacticus* and the organisms associated with it in starters. Agr. Exp. Sta. Iowa Res. Bull. 63, 59–96.

[B51] Hill C. Guarner F. Reid G. Gibson G. R. Merenstein D. J. Pot B.. (2014). Expert consensus document: The International Scientific Association for Probiotics and Prebiotics consensus statement on the scope and appropriate use of the term probiotic. Nat. Rev. Gastroenterol. Hepatol. 11, 506–514. 10.1038/nrgastro.2014.66 24912386

[B52] Hillion M. Mijouin L. Jaouen T. Barreau M. Meunier P. Lefeuvre L.. (2013). Comparative study of normal and sensitive skin aerobic bacterial populations. Microbiologyopen 2, 953–961. 10.1002/mbo3.138 24151137 PMC3892341

[B53] Holzapfel W. H. Franz C. M. A. P. Ludwig W. Back W. Dicks L. M. T. (2006). The genera *Pediococcus* and *Tetragenococcus*, in The Prokaryotes, eds Dworkin M. F. S. Rosenberg E. Schleifer K. H. Stackebrandt E. E (New York, NY: Springer-Verlag), 229–266. 10.1007/0-387-30744-3_8

[B54] Holzapfel W. H. Wood B. J. B. H. (2014). Lactic Acid Bacteria: Biodiversity and Taxonomy, 1st Edn. Somerset: Wiley.

[B55] Humble M. W. King A. Phillips I. (1977). API ZYM: a simple rapid system for the detection of bacterial enzymes. J. Clin. Pathol. 30, 275–277. 10.1136/jcp.30.3.275 845275 PMC476372

[B56] Ilavenil S. Vijayakumar M. Kim D. H. Valan Arasu M. Park H. S. Ravikumar S.. (2016). Assessment of probiotic, antifungal and cholesterol lowering properties of *Pediococcus pentosaceus* KCC-23 isolated from Italian ryegrass. J. Sci. Food Agric. 96, 593– 601. 10.1002/jsfa.7128 25655225

[B57] Jamuna M. Jeevaratnam K. (2004). Isolation and partial characterization of bacteriocins from *Pediococcus* species. Appl. Microbiol. Biotechnol. 65, 433–439. 10.1007/s00253-004-1576-8 15205931

[B58] Johansson D. Rasmussen M. (2013). Virulence factors in isolates of *Enterococcus faecalis* from infective endocarditis and from the normal flora. Microb. Pathog. 55, 28–31. 10.1016/j.micpath.2012.09.009 23044056

[B59] Kim J. W. Rajagopal S. N. (2001). Antibacterial activities of *Lactobacillus crispatus* ATCC 33820 and *Lactobacillus gasseri* ATCC 33323. J. Microbiol. 39, 146–148.

[B60] Klingspor S. Bondzio A. Martens H. Aschenbach J. R. Bratz K. Tedin K.. (2015). *Enterococcus faecium* NCIMB 10415 modulates epithelial integrity, heat shock protein, and proinflammatory cytokine response in intestinal cells. Mediators Inflamm. 2015:304149 10.1155/2015/304149 25948884 PMC4408629

[B61] Ladha G. Jeevaratnam K. (2018). Probiotic potential of *Pediococcus pentosaceus* LJR1, a bacteriocinogenic strain isolated from rumen liquor of goat (*Capra aegagrus* hircus). Food Biotechnol. 32, 60–77. 10.1080/08905436.2017.1414700

[B62] Le Marrec C. Hyronimus B. Bressollier P. Verneuil B. Urdaci M. C. (2000). Biochemical and genetic characterization of coagulin, a new antilisterial bacteriocin in the pediocin family of bacteriocins, produced by Bacillus coagulans I(4). Appl. Environ. Microbiol. 66, 5213–5220. 11097892 10.1128/aem.66.12.5213-5220.2000 PMC92446

[B63] Lee K. W. Park J. Y. Sa H. D. Jeong J. H. Jin D. E. Heo H. J.. (2014). Probiotic properties of *Pediococcus* strains isolated from jeotgals, salted and fermented Korean sea-food. Anaerobe 28, 199–206. 10.1016/j.anaerobe.2014.06.013 24979684

[B64] Lv L. X. Li Y. D. Hu X. J. Shi H. Y. Li L. J. (2014). Whole-genome sequence assembly of *Pediococcus pentosaceus* LI05 (CGMCC 7049) from the human gastrointestinal tract and comparative analysis with representative sequences from three food-borne strains. Gut Pathog. 6:36. 10.1186/s13099-014-0036-y 25349631 PMC4209512

[B65] Mannu L. Paba A. Daga E. Comunian R. Zanetti S. Dupr,è I.. (2003). Comparison of the incidence of virulence determinants and antibiotic resistance between *Enterococcus faecium* strains of dairy, animal and clinical origin. Int. J. Food. Microbiol. 88, 291–304. 10.1016/S0168-1605(03)00191-0 14597001

[B66] Marchesi J. R. Sato T. Weightman A. J. Martin T. A. Fry J. C. Hiom S. J.. (1998). Design and evaluation of useful bacterium-specific PCR primers that amplify genes coding for bacterial 16S rRNA. Appl. Environ. Microbiol. 64, 795–799. 9464425 10.1128/aem.64.2.795-799.1998 PMC106123

[B67] Martino M. E. Maifreni M. Marino M. Bartolomeoli I. Carraro L. Fasolato L. (2013). Genotypic and phenotypic diversity of *Pediococcus pentosaceus* strains isolated from food matrices and characterization of penocin operon. Antonie Van Leeuwenhoek. 103, 1149–1163. 10.1007/s10482-013-9897-1 23444039

[B68] Marugg J. D. Gonzalez C. F. Kunka B. S. Ledeboer A. M. Pucci M. J. Toonen M. Y.. (1992). Cloning, expression, and nucleotide sequence of genes involved in production of pediocin PA-1, a bacteriocin from *Pediococcus acidilactici* PAC1.0. Appl. Environ. Microbiol. 58, 2360–2367. 1514784 10.1128/aem.58.8.2360-2367.1992 PMC195787

[B69] Mathys S. von Ah U. Lacroix C. Staub E. Mini R. Cereghetti T.. (2007). Detection of the pediocin gene pedA in strains from human faeces by real-time PCR and characterization of *Pediococcus acidilactici* UVA1. BMC Biotechnol. 7:55. 10.1186/1472-6750-7-55 17850651 PMC2034554

[B70] Mazzoli R. Bosco F. Mizrahi I. Bayer E. A. Pessione E. (2014). Towards lactic acid bacteria-based biorefineries. Biotechnol. Adv. 32, 1216–1236. 10.1016/j.biotechadv.2014.07.005 25087936

[B71] Midha S. Ranjan M. Sharma V. Kumari A. Singh P. K. Korpole S. (2012). Genome sequence of *Pediococcus pentosaceus* strain IE-3. J. Bacteriol. 194:4468 10.1128/JB.00897-12 22843596 PMC3416253

[B72] Monteagudo-Mera A. Rodriguez-Aparicio L. Rua J. Martinez-Blanco H. Navasa N. Garcia-Armesto M. R. (2012). *In vitro* evaluation of physiological probiotic properties of different lactic acid bacteria strains of dairy and human origin. J. Funct. Foods. 4, 531–541. 10.1016/j.jff.2012.02.014

[B73] Moraes P. M. Perin L. M. Ortolani M. B. T. Yamazi A. K. Vicosa G. N. Nero L. A. (2010). Protocols for the isolation and detection of lactic acid bacteria with bacteriocinogenic potential. LWT- Food Sci. Technol. 43, 1320–1324. 10.1016/j.lwt.2010.05.005

[B74] Morandi S. Brasca M. Andrighetto C. Lombardi A. Lodi R. (2006). Technological and molecular characterization of *Enterococci* isolated from NorthWest Italian dairy products. Int. Dairy J. 16, 867–875. 10.1016/j.idairyj.2005.09.005

[B75] Motlagh A. M. Bhunia A. K. Szostek F. Hansen T. R. Johnson M. C. Ray B. (1992). Nucleotide and amino acid sequence of pap-gene (pediocin AcH production) in *Pediococcus acidilactici* H. Lett. Appl. Microbiol. 15, 45–48. 10.1111/j.1472-765X.1992.tb00721.x 1368421

[B76] Muñoz-Atienza E. Gomez-Sala B. Araujo C. Campanero C. del Campo R. Hernández P. E.. (2013). Antimicrobial activity, antibiotic susceptibility and virulence factors of Lactic Acid Bacteria of aquatic origin intended for use as probiotics in aquaculture. BMC Microbiol. 13:15. 10.1186/1471-2180-13-15 23347637 PMC3574848

[B77] Narayanan R. Sumanth G. K. Chowdary R. Jyothi C. P. (2017). *In Vitro* study of potential probiotic *Pediococcus pentosaceus* isolated from Idli batter and biomass production using whey. Int. J. Food Nutr. Sci. 6, 34–45.

[B78] Noohi N. Ebrahimipour G. Rohani M. Talebi M. Pourshafie M. R. (2016). Evaluation of potential probiotic characteristics and antibacterial effects of strains of *Pediococcus* species isolated from broiler chickens. Br. Poult. Sci. 57:317–323. 10.1080/00071668.2016.1169247 27057800

[B79] Oh Y. J. Jung D. S. (2015). Evaluation of probiotic properties of *Lactobacillus* and *Pediococcus* strains isolated from Omegisool, a traditionally fermented millet alcoholic beverage in Korea. LWT - Food Sci. Technol. 63, 437–444. 10.1016/j.lwt.2015.03.005

[B80] Osmanagaoglu O. Kiran F. Ataoglu H. (2010). Evaluation of *in vitro* probiotic potential of *Pediococcus pentosaceus* OZF isolated from human breast milk. Probiotics Antimicrob. Proteins 2, 162–174. 10.1007/s12602-010-9050-7 26781239

[B81] Papagianni M. (2003). Ribosomally synthesized peptides with antimicrobial properties: biosynthesis, structure, function, and applications. Biotechnol. Adv. 21, 465–499. 10.1016/S0734-9750(03)00077-6 14499150

[B82] Papagianni M. (2012). Metabolic engineering of lactic acid bacteria for the production of industrially important compounds. Comput. Struct. Biotechnol. J. 3:e201210003. 10.5936/csbj.201210003 24688663 PMC3962192

[B83] Papagianni M. Anastasiadou S. (2009). Pediocins: the bacteriocins of *Pediococci*. Sources, production, properties and applications. Microb. Cell Fact. 8, 3–18. 10.1186/1475-2859-8-3 19133115 PMC2634753

[B84] Piva A. Headon D. R. (1994). Pediocin A, a bacteriocin produced by *Pediococcus pentosaceus* FBB61. Microbiology 140, 697–702. 10.1099/00221287-140-4-697 8012591

[B85] Puniya M. Ravinder Kumar M. Panwar H. Kumar N. Ramneek Anil Kumar P. (2016). Screening of lactic acid bacteria of different origin for their probiotic potential. J. Food Process Technol. 7:1 10.4172/2157-7110.1000545

[B86] Resta-Lenert S. Barrett K. E. (2003). Live probiotics protect intestinal epithelial cells from the effects of infection with enteroinvasive *Escherichia coli* (EIEC). Gut 52, 988–997. 10.1136/gut.52.7.988 12801956 PMC1773702

[B87] Ríos Colombo N. S. Chalón M. C. Navarro S. A. Bellomio A. (2017). Pediocin-like bacteriocins: new perspectives on mechanism of action and immunity. Curr. Genet. 64, 345–351. 10.1007/s00294-017-0757-9 28983718

[B88] Rodriguez J. M. Cintas L. M. Casaus P. Martinez M. I. Suarez A. Hernandez P. E. (1997). Detection of pediocin PA-1-producing pediococci by rapid molecular biology techniques. Food Microbiol. 14, 363–371. 10.1006/fmic.1996.0084

[B89] Rodríguez J. M. Martinez M. I. Kok J. (2002). Pediocin PA-1, a wide-spectrum bacteriocin from lactic acid bacteria. Crit. Rev. Food Sci. Nutr. 42, 91–121. 10.1080/10408690290825475 11934133

[B90] Rosenberg M. Gutnick D. Rosenberg E. (1980). Adherence of bacteria to hydrocarbons: a simple method for measuring cell-surface hydrophobicity. FEMS Microbiol. Lett. 9, 29–33 10.1111/j.1574-6968.1980.tb05599.x

[B91] Ryu E. H. Chang H. C. (2013). *In vitro* study of potentially probiotic lactic acid bacteria strains isolated from Kimchi. Ann. Microbiol. 63, 1387–1395. 10.1007/s13213-013-0599-8

[B92] Saad N. Delattre C. Urdaci M. Schmitter J. M. Bressollier P. (2013). An overview of the last advances in probiotic and prebiotic field. LWT Food Sci. Technol. 50, 1–16. 10.1016/j.lwt.2012.05.014

[B93] Savedboworn W. Riansa-Ngawong W. Sinlapacharoen W. Pajakang S. Patcharajarukit B. Tipkanon S. (2014). Assessment of probiotic properties in lactic acid bacteria isolated from fermented vegetables. Int. J. Appl. Sci. Technol. 7, 53–65. 10.14416/j.ijast.2014.10.001

[B94] Schnurer J. Magnusson J. (2005). Antifungal lactic acid bacteria as biopreservatives. Trends Food Sci. Technol. 16, 70–78. 10.1016/j.tifs.2004.02.014

[B95] Schuppler M. Loessner M. (2010). The opportunistic pathogen *Listeria monocytogenes*: pathogenicity and interaction with the mucosal immune system. Int. J. Inflam. 2010:704321 10.4061/2010/704321 21188219 PMC3003996

[B96] Semjonovs P. Zikmanis P. (2008). Evaluation of novel lactose-positive and exopolysaccharide-producing strain of *Pediococcus pentosaceus* for fermented foods. Eur. Food Res. Technol. 227, 851–856. 10.1007/s00217-007-0796-4

[B97] Sharma P. Tomar S. K. Goswami P. Sangwan V. Singh R. (2014). Antibiotic resistance among commercially available probiotics. Food Res. Int. 57, 176–195. 10.1016/j.foodres.2014.01.025

[B98] Sherman P. M. Johnson-Henry K. C. Yeung H. P. Ngo P. S. C. Goulet J. Tompkins T. A. (2005). Probiotics reduce enterohemorrhagic *Escherichia coli* O157:H7 - and enteropathogenic *E. coli* O127:H6-induced changes in polarized T84 epithelial cell monolayers by reducing bacterial adhesion and cytoskeletal rearrangements. Infect. Immun. 73, 5183–5188. 10.1128/IAI.73.8.5183-5188.2005 16041036 PMC1201237

[B99] Shi Y. Cui X. Gu S. Yan X. Li R. Xia S.. (2018). Antioxidative and probiotic activities of lactic acid bacteria isolated from traditional artisanal milk cheese from Northeast China. Probiotics Antimicrob. Prot. [Epub ahead of print]. 10.1007/s12602-018-9452-5 30056601

[B100] Shukla R. Goyal A. (2014). Probiotic potential of *Pediococcus pentosaceus* CRAG3: a new isolate from fermented cucumber. Probiotics Antimicrob. Prot. 6, 11–21. 10.1007/s12602-013-9149-8 24676763

[B101] Sogawa K. Watanabe M. Sato K. Segawa S. Ishii C. Miyabe A.. (2011). Use of the MALDI BioTyper system with MALDI-TOF mass spectrometry for rapid identification of microorganisms. Anal. Bioanal. Chem. 400, 1905–1911. 10.1007/s00216-011-4877-7 21442367

[B102] Swaminathan B. Gerner-Smidt P. (2007). The epidemiology of human listeriosis. Microb. Infect. 9, 1236–1243. 10.1016/j.micinf.2007.05.011 17720602

[B103] Tambekar D. H. Bhutada S. A. Choudhary S. D. Khond M. D. (2009). Assessment of potential probiotic bacteria isolated from milk of domestic animals. J. Appl. Biosci. 15, 815–819.

[B104] Temmerman R. Pot B. Huys G. Swings J. (2003). Identification and antibiotic susceptibility of bacterial isolates from probiotic products. Int. J. Food Microbiol. 81, 1–10. 10.1016/S0168-1605(02)00162-9 12423913

[B105] Todorov S. D. Dicks L. M. (2009). Bacteriocin production by *Pediococcus pentosaceus* isolated from marula (*Scerocarya birrea*). Int. J. Food Microbiol. 132, 117–126. 10.1016/j.ijfoodmicro.2009.04.010 19446352

[B106] Toomey N. Bolton D. Fanning S. (2010). Characterization and transferability of antibiotic resistance genes from lactic acid bacteria isolated from Irish pork and beef abattoirs. Res. Microbiol. 161, 127–135. 10.1016/j.resmic.2009.12.010 20074643

[B107] Vidhyasagar V. Jeevaratnam K. (2012). Evaluation of *Pediococcus pentosaceus* strains isolated from Idly batter for probiotic properties *in vitro*. J. Funct. Foods 5, 235–243. 10.1016/j.jff.2012.10.012

[B108] Wang C. Y. Lin P. R. Ng C. C. Shyu Y. T. (2010). Probiotic properties of *Lactobacillus* strains isolated from the feces of breast-fed infants and Taiwanese pickled cabbage. Anaerobe 16, 578–585. 10.1016/j.anaerobe.2010.10.003 20951815

[B109] Zommiti M. Cambronel M. Maillot O. Barreau M. Sebei K. Feuilloley M. G.. (2018). Evaluation of probiotic properties and safety of *Enterococcus faecium* isolated from artisanal Tunisian meat ‘Dried Ossban'. Front. Microbiol. 9:1685. 10.3389/fmicb.2018.01685 30127770 PMC6088202

