# Supplementary figures and images for: In vitro Assessment of the Probiotic Properties and Bacteriocinogenic Potential of Pediococcus pentosaceus MZF16 Isolated From Artisanal Tunisian Meat “Dried Ossban”
Source: Front Microbiol. 2018 Nov 9;9:2607. doi: 10.3389/fmicb.2018.02607 (PMC6238632; doi:10.3389/fmicb.2018.02607)

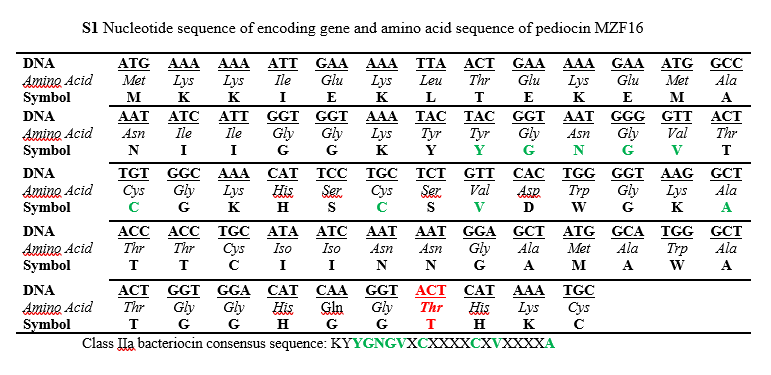

Supplement: Supplementary file 1 [file Image_1.TIF]
